# Transmission dynamics of SARS-CoV-2 in a mid-size city of China

**DOI:** 10.1186/s12879-021-06522-9

**Published:** 2021-08-10

**Authors:** Hongjun Zhao, Xiaoxiao Lu, Wenhui Lun, Tiegang Li, Boqi Rao, Dedong Wang, Di Wu, Fuman Qiu, Zhicong Yang, Jiachun Lu

**Affiliations:** 1 State Key Lab of Respiratory Disease, The First Affiliated Hospital, Institute for Public Health, School of Public Health, Guangzhou Medical University, 195 Dongfengxi Road, Guangzhou, 510182 China; 2 Department of English and American Studies, Faculty of Languages and Literatures, Ludwig Maximilian University (LMU), Munich, Germany; 3 Department of Epidemiology, Guangzhou Chest Hospital, No. 62 Hengzhigang Road, Guangzhou, 510095 China; 4 Guangzhou Centre for Disease Control and Prevention, No. 1 Qide Road, Guangzhou, 510440 China

**Keywords:** SARS-CoV-2, COVID-19, Asymptomatic patient, Transmission dynamics, R_0_, R_t_, Prevention and control measures

## Abstract

**Background:**

An outbreak of pneumonia, COVID-19 associated with the severe acute respiratory syndrome coronavirus 2 (SARS-CoV-2) emerged in Wuhan city and then rapidly spread to other cities. Wenzhou is located approximately 900 km from Wuhan, which was experiencing an outbreak that was severe at the time but is considered modest as the epidemic became a pandemic. We described the epidemiological characteristics of SARS-CoV-2 outside of the epicenter to help understand the transmission pattern in a mid-sized Chinese city.

**Methods:**

To investigate the epidemiological and clinical characteristics of the COVID-19, we described case series of 473 patients with confirmed COVID-19 in Wenzhou, China from January 27 to March 16, 2020. We described the public health interventions of COVID-19 and evaluated the effect of interventions by the effective reproduction number (R_t_).

**Results:**

The median age of all patients was 47.6 years, 48.4% of whom were female. 33.8% of the patients had a history of residence in Wuhan. Fever (71.7%) and cough (43.1%) were the most common symptoms. In addition, three kinds of unconventional cases were observed, namely 4.9% asymptomatic patients, 7.6% confirmed patients who had no link to Wuhan city but contact with individuals from Wuhan without any symptoms at the time of contact, and 12.9% confirmed patients who had an unknown source of transmission. We estimated that the basic reproductive number (R_0_) was 2.75 (95% CI: 2.37–3.23). The R_t_ fluctuated within the range of 2.50 to 3.74 from January 11 to January 16 while gradually reached a peak of 3.74 on January 16. R_t_ gradually decreased after January 16 and decreased to 1.00 on January 30. R_t_ continually decreased and reached the lowest point (0.03) on February 21, 2020.

**Conclusion:**

Our study presented the possibility of asymptomatic carriers affected with SARS-CoV-2, and transmission by these three kinds of unconventional patients in Wenzhou may be an important characteristic of SARS-CoV-2 transmission. The evaluation showed that a series of multifaceted interventions proved effective in controlling the epidemic of COVID-19. These findings might provide valuable examples of control policies for countries or areas in combatting the global pandemic of COVID-19.

**Supplementary Information:**

The online version contains supplementary material available at 10.1186/s12879-021-06522-9.

## Background

It has been ten months since threatening pneumonia, COVID-19 infected by the severe acute respiratory syndrome coronavirus 2 (SARS-CoV-2) was first reported in Wuhan, China on December 31, 2019 [1, 2]. In the first few months, the COVID-19 epidemic had spread to other Chinese cities at fast speed [3–5]. An raising number of epidemiological evidence suggested the existence of person-to-person transmission in hospitals and in household [6–8], showing an overwhelming challenge for control of this novel coronavirus pneumonia (NCP).

The World Health Organization had declared this NCP as a “Public Health Emergency of International Concern” (PHEIC) and officially termed it Corona Virus Disease 2019 (COVID-19). As of March 16, 2020, the Chinese government reported 80,881 confirmed cases in mainland China [9]. Apart from the Wuhan city area, which includes the initial epidemic city and its surrounding cities, other major cities in China and other countries soon experienced localized outbreaks [10, 11]. Huang et al. first reported on the cases of COVID-19, among which most patients had a history of exposure in a Seafood Wholesale Market [2]. Wang et al. also identified that patients with COVID-19 shared common symptoms including fever, fatigue, and dry cough [1].

As of March 16, in areas outside of Hubei Province, the slowdown in the increase rate of confirmed patients and suspected patients also indicated that the measures and strategies of prevention and control had proven effective [12]. Wenzhou (a mid-city with 8 million residents) is a prefecture-level city of Zhejiang province and has been identified as an epidemic area because it is one of the Chinese cities with a large number of confirmed cases outside of Wuhan. There are many people of this city engaging in trade and businessmen of this city are famous for being distributed throughout China. It was critical that many people returned to this city from Wuhan before the lockdown because of the Spring Festival. There was also evidence indicating that the epidemic situation in some cities had shifted from the import stage to the local community spread stage. In some countries or cities, widespread community transmission had occurred due to the failure or delay in implementing effective prevention and control strategies of COVID-19 while the epidemic was under control after implementing a series of interventions in Wenzhou, China.

## Methods

### Data collection

Epidemiological surveys were conduct on data collected from laboratory-confirmed cases, including age, gender, epidemiological history, clinical symptoms and outcome, extracting from the Municipal Health Commission.

(Website: http://wjw.wenzhou.gov.cn/col/col1209919/index.html).

### Study definitions

Since the early stage of COVID-19 outbreak, many monitoring strategies and interventions were implemented across Wenzhou to detect suspected and confirmed COVID-19 cases as well as their close contacts following standardized protocols released by the National Health Commission of China. In our study, the definitions of the COVID-19 patient, the severe case, the asymptomatic patient, the exposure, cluster outbreak, the suspected case and co-exposed person were based on the 6th edition of the Diagnosis and Treatment Scheme by the National Health Commission of China and the definition of close contact were based on the 5th edition of the Prevention and Control Scheme by the National Health Commission of China [13, 14]. The unconventional patients and the conventional patients were defined specifically by the authors of this study based on the study purpose.

The COVID-19 patient was defined as an individual with a positive result of real-time reverse-transcriptase–polymerase-chain-reaction (RT-PCR) assay of the SARS-CoV-2 virus in pharyngeal and anal swab specimens. Only the laboratory-confirmed patients were included in the final analysis [13, 37].

The severe patient was defined as patient who meet any of the following criteria: (1) dyspnea or respiratory rate ≥ 30 breaths/min; (2) oxygen saturation ≤ 93% at a rest state; (3) arterial partial pressure of oxygen (PaO2)/oxygen concentration (FiO2) ≤ 300 mmHg; (4) Patients with > 50% lesions progression within 24 to 48 h in lung imaging should be regarded as severe cases [14].

The asymptomatic patient was defined as a patient who showed no clinical symptoms at the time of testing (and 14 days before the testing) but had a positive result of SARS-CoV-2 on RT-PCR [13, 37]. The definition of exposure, cluster outbreak, close contacts, suspected cases and co-exposed person are shown in Additional Methods in Additional file 1: Appendix S1.

The unconventional patients included asymptomatic patients, and patients who had no link to Wuhan city but had contact with individuals from Wuhan without any symptoms at the time of contact, and patients who had an unknown source of transmission.

The conventional patients were those who had a known source of transmission and symptoms.

### Laboratory confirmation

Laboratory confirmation of the SARS-CoV-2 by RT-PCR assay was conducted in Wenzhou Center for Disease Prevention and Control (WZCDC) [14]. The RT-PCR assay was performed following the protocol established by the World Health Organization [15]. SARS-CoV-2 nucleic acid testing was performed using RT-PCR assay according to the National Health Commission guidelines for laboratory testing of pneumonia with novel coronavirus infection (2nd Edition, 3rd Edition) [2].

### Statistical analysis

Continuous variables were presented using the mean and standard deviation or median and interquartile range (IQR) as appropriate, and categorical variables were described as frequency rates and percentages.

R_0_ was defined as the expected number of additional cases that one case will generate on average, over the course of its infections period in other uninfected population. We estimated R_0_ on the cases with symptom onset between January 9 and January 22, during which we expected the number of infections to soon increase in Wenzhou. R_t_ was defined as the mean number of secondary cases generated by a typical primary case at time t in a population [16]. We estimated R_t_ and its 95% credible interval on each day from January 11 to March 2 via a 7-day moving average. We used the serial interval with a mean of 7.5 and a standard deviation of 3.4 based on the study of Li et al. [17]. The analyses of R_0_ and R_t_ were conducted by using the R_0_ and EpiEstim packages in the R statistical programming language, version 3.6.3 (R Foundation for Statistical Computing). Other analyses were performed using SPSS software (Statistical Package for the Social Sciences) version 26.0 (SPSS Inc). All the figures were drawn using GraphPad Prism 8 software.

## Results

### Clinical and epidemiological characteristics

**Fig. 1:**
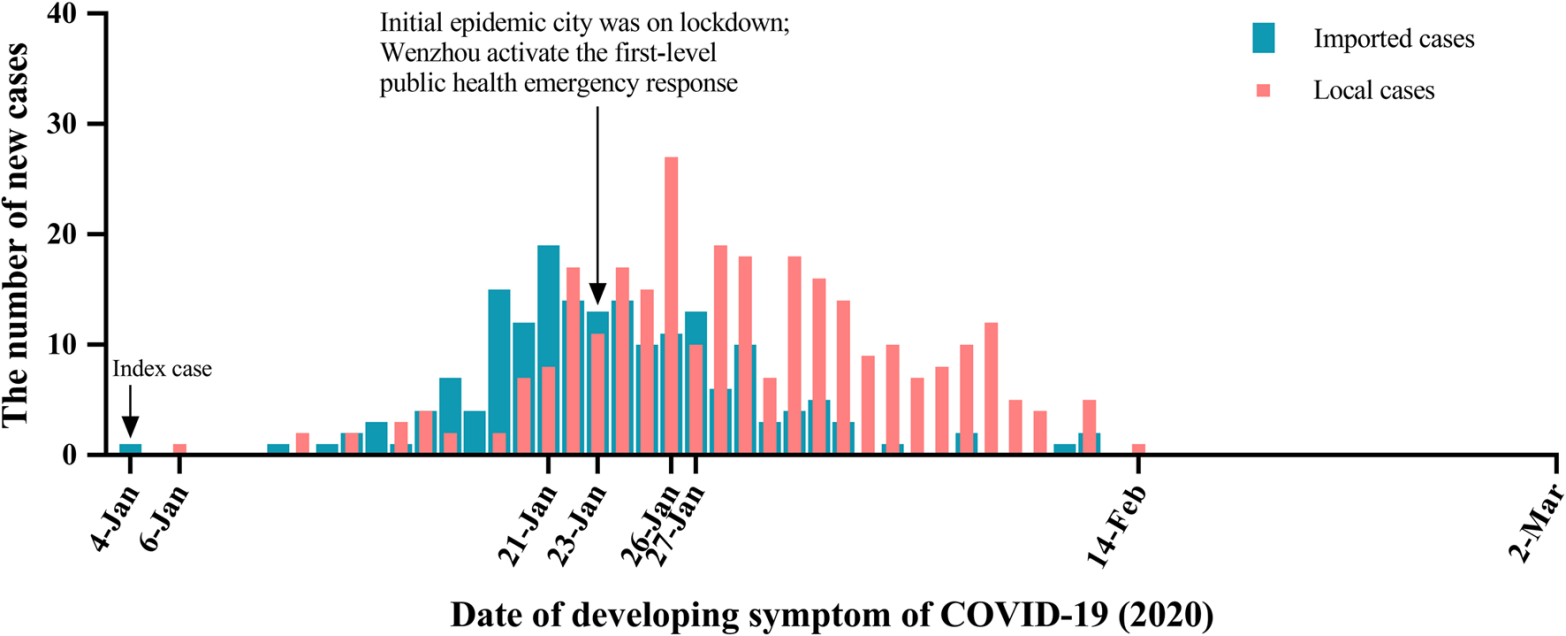
The time distribution of imported and local cases

As shown in Table 1 and Fig. 1, of all 504 patients reported as of March 16 in Wenzhou, China, 31(6.2%) patients were excluded due to the incomplete status of the information (including clinical outcomes and symptoms) by the publishing date, and 473(93.8%) patients had completed characteristics. The median age was 47.6 years (IQR, 37–56; range, 2–93 years), and 222 (48.4%) were females. The median time from onset to diagnosis was 6.0 days (range: 0–23 days). Fever (71.7%) and cough (43.1%) were the most common symptoms, whereas diarrhea or appetite loss (3.2%) were rare. 160 (33.8%) patients had a history of travel or residence to Wuhan. 51 (10.8%) were severe patients and 422 (89.2%) were non-severe patients. There were 472 cases were recovery and discharged and 1 death occurred in Wenzhou as of March 16.

**Table 1:** Clinical and epidemiological characteristics of the 473 COVID-19 patients in Wenzhou, China

Early clinical symptoms	Wenzhou
	n/ (%)
Female sex	222 (48.4)
Age, Median (range)—years	47.6 (2–93)
Travel or residence history within 14 days	
Recently been to Wuhan	160 (33.8)
Outside Wuhan or Wenzhou in China	24 (5.1)
Wenzhou	289 (61.1)
Contact with source of transmission within 14 days	
Contacted with people from Wuhan	36 (7.6)
Contacted with patient	195 (41.2)
Related the Yintai world trade center	17 (3.6)
Undiscovered source of transmission	61 (12.9)
Unknown	164 (34.7)
Symptoms	
Asymptoms	23 (4.9)
Symptoms	
Fever	339 (71.7)
Headache	31 (6.6)
Cough	204 (43.1)
Sore throat	39 (8.2)
Sputum production	50 (10.6)
Fatigue	57 (12.1)
Diarrhea or appetite loss	15 (3.2)
Muscle soreness	29 (6.1)
Disease severity	
Severe	51 (10.8)
Clinical outcomes	
Discharge from hospital	472 (99.8)
Death	1 (0.2)
Time from onset to diagnosis (days)	
Median (range)	6.0 (0–23)

313 cases that had no link to Wuhan accounted for 66.2% of 473 cases and 160 cases with links to Wuhan accounted for 33.8% of the total. We described the epidemic curve of onset dates for both imported and local cases. As shown in Fig. 1, we could see the change in the daily number of new cases of imported cases and local cases. The earliest onset time of the imported cases was January 4, with a peak on January 21. The number of new cases gradually reduced after January 27. The local cases started to onset on January 6, numbers peaked on January 26 and decreased significantly after that. No case occurred in Wenzhou after February 14.

### Features of three kinds of unconventional COVID-19 cases

The point worth noting about COVID-19 in Wenzhou was the emergence of three kinds of unconventional cases. As shown in Table 1, there were 23 asymptomatic carriers accounts for 4.9% of the total patients, 36 (7.6%) patients who had no link to Wuhan city but contact with individuals from Wuhan without any symptoms at the time of contact and 61 (12.9%) patients who had an unknown source of transmission.

### A potential super-spreader event

Moreover, there was an outbreak of infection in a public place at the Yintai world trade center. On January 20, a 39-year-old saleswoman went to a local hospital for treatment by herself after breaking out with a fever at 38.5℃, accompanied by chills, dizziness, and headache, and symptoms of soreness and fatigue. She was subsequently diagnosed with COVID-19 on January 28, but the source of which was unknown. Eventually, a total of 16 additional patients resulting from a contact within this mall had been confirmed, namely two staff members, two salesmen, one janitor, nine customers, and two individuals outside the mall but with close contact with one of the above.

## Intervention and surveillance measures

### Early-stage without strong interventions

The intervention and surveillance measures of COVID-19 were shown in Table 2. On January 21, 2020, the Wenzhou government publicly announced the 24-h telephone hotline of CDC to provide COVID-19 medical consultation for citizens in Wenzhou. At the same time, Wenzhou government mobilized community workers to conduct the follow-up survey for people from Wuhan. Moreover, Wenzhou government had started implementing temperature monitoring in transportation hubs and disinfection in critical public places [18, 19].

**Table 2:** Public health interventions across the 3 stage during the COVID-19 outbreak in Wenzhou, China

Interventions			Starting date
Control the source of infection	Cut the transmission routes	Prevent new infections	
Early stage without strong interventions (Before January 23th)			
Conducting survey for people from Wuhan; Temperature monitoring in transportation hubs	Disinfection in public	Personal health education	1/21
Establishment of self-reporting channels; Designated fever clinical institution	Cancellation of public events; Cancellation of wildlife trading	–	1/22
–	–	–	
Intensification stage of COVID-19 interventions (January 23th to February 15th)			
Quarantine of people from Wuhan and their close contacts	Cancellation of gatherings; Closures of public venues	Requirement of wearing mask and washing hands	1/24
Providing hotlines for self-reporting	-	–	1/26
Quarantine of people from Hubei and their close contacts;	Suspension of inter-city traffic; Delaying the resumption of work and school; Implementing closed-off management in nursing Home	–	1/27
–	Suspension of highways, railways and ferries	–	1/29
–	-	Home quarantine for all citizen	2/1 – 2/15
Normalization mode of COVID-19 epidemic prevention and control (After February 16th)			
Quarantine of people from high-risk areas and their close contacts;	Close-loop and Close-off management	“Wenzhou Health Code”	2/16
–	Resumption of work and production; Reopened the highway	–	2/20 – 2/22
–	Reopened of public venue (Except of indoor places)	–	3/2

On January 22, 2020, Wenzhou government cancelled all public events and banned the wildlife trading. It implemented a daily and zero-reporting system for COVID-19 cases by Direct Network Report systems of Infectious Diseases and established proactive self-reporting channels for people returning from Wuhan and those with symptoms. Meanwhile, it publicly announced 33 24-h fever clinical institutions and 9 designated medical institutions [19, 20].

### Intensification stage of COVID-19 interventions

On January 23, 2020, the Wenzhou government launched the first-level response to major public health emergencies [21], and implemented a serial of stricter interventions on January 24. It suspended live poultry trading, cancelled all large gatherings, and closed public entertainment venues. Body temperature testing and daily disinfection were implemented in all public places. Individuals in Wenzhou must wear masks when they went out. People who came to Wenzhou from Wuhan and their close contacts were required to quarantine at home for 14 days. If they had a fever, especially persistent fever or respiratory symptoms, they must go to the local fever clinic in time [21]. On January 26, 2020, the Wenzhou government set up a 24-h public telephone hotline for citizens to encourage active self-report who had recently come to Wenzhou and had not undergone medical isolation [22].

On January 27, 2020, people from Hubei Province, close contacts, and fever cases were required to self-quarantine at home or designated locations for 14 days. Inter-provincial and inter-city shuttle buses and chartered passenger transport were suspended. The essential public places, like supermarkets, were required to reasonably adjust business hours and regularly have disinfection. Nursing homes were required to temporarily implement closed management. All colleges, primary and secondary schools were forbidden to start the new semester on schedule while all kinds of extracurricular training activities were also put on hold. Wenzhou government delayed the resumption of work [23].

On January 29, 2020, 14 highway intersections entering, railways and all ferries in Wenzhou were suspended [24]. From February 1 to February 15, 2020, Wenzhou introduced a regulation that only one person in each household could go out to purchase every 2 days [25, 26].

### Normalization mode of COVID-19 epidemic prevention and control

Starting from February 16, 2020, a normalization mode of the epidemic prevention and control was implemented and the "Wenzhou Health Code" (Additional file 1: Appendix S1) was promoted and applied throughout the city. Villages and communities continued to implement closed-loop management (Additional file 1: Appendix S1). But when a cluster of cases broke out, the community must immediately implement full or partial closed-off management (Additional file 1: Appendix S1) according to the risk level [27, 28].

Wenzhou citizen and people from other areas who were ready to return to Wenzhou could apply online for "Wenzhou Health Code" based on their health status and travel history. A three-color "health code" would be generated, including green, yellow and red code. Those who displayed a "green code" and whose temperature measurement was normal were allowed to pass while those who displayed a "yellow code" would be subject to 14-day and 7-day home quarantine observation according to the travel history of epidemic-risk areas and low-risk areas respectively. Those that displayed the "red code" were sent to the designated hospital for treatment or medical isolation and observation for 14 days.

From February 20 to 22, 2020, work and production activities gradually resumed in Wenzhou. The relevant checkpoints were removed and the highway entrances and exits were simultaneously reopened [29].

### Evaluation of effects for prevention and control measures

We estimated that the R0 to be 2.75 (95% CI: 2.37–3.23) for the cases with symptom onset between January 9 and January 22 using the Exponential Growth method, thus we expected the proportion of infections to increase thereafter in Wenzhou. The results showed that in the early stage of the epidemic, COVID-19 in Wenzhou had strong infectivity, consistent with results of previous studies [17].

**Fig. 2:**
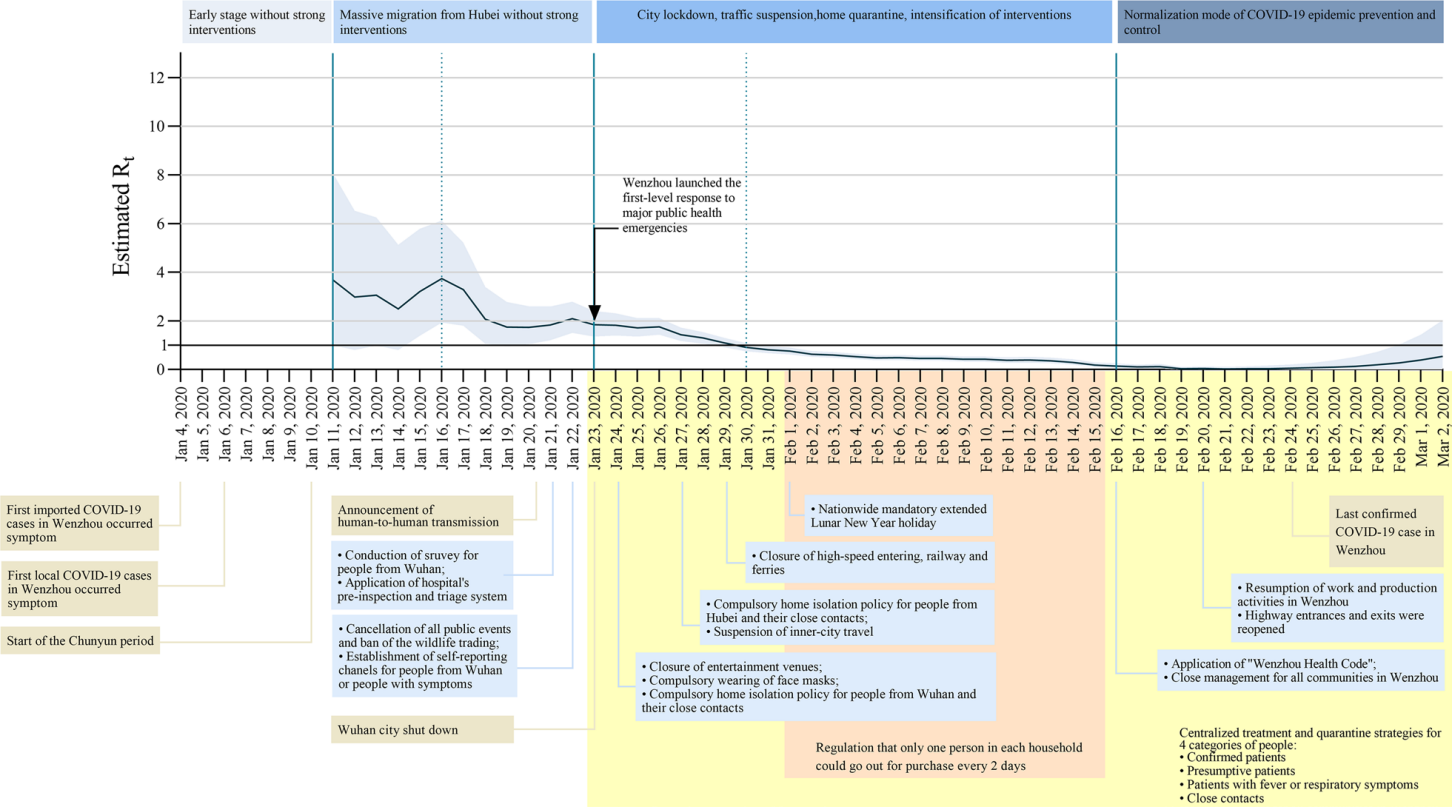
The effective reproduction number estimates based on laboratory-confirmed COVID-19 cases and public health control measures in Wenzhou. Results were shown since January 11, calculated for the whole period (from January 11 to March 2) over 7-day moving average. The black horizontal line indicated R_t_ = 1, below which sustained transmission is unlikely so long as public health control measures were sustained, indicating that the outbreak is under control. The 95% credible intervals (CI) were presented as light orange shading. Daily estimates of R_t_ with 95% CrIs were shown in Additional file 2: Table S1

Based on the study of Li et al. [17], we estimated R_t_ of Wenzhou from January 11 to March 2, 2020, with a 7-day moving average, which was an index to evaluate the effect of prevention and control strategy. R_t_ fluctuated within the range of 2.50 to 3.74 from January 11 to January 16 while gradually reached a peak of 3.74 on January 16. R_t_ decreased after January 16 to 1.00 on January 30. R_t_ continually decreased and reached the lowest point (0.03) on February 21. Though the trend of R_t_ was slightly increasing between February 22 to March 2, the estimation of R_t_ still below 0.38. The daily estimated values of R_t_ were shown in Additional file 2: Table **S**1 and Fig. 2.

#### Discussion

In this study, the declination of the number of COVID-19 incidences and R_t_ indicated that the COVID-19 outbreak in Wenzhou was under control after implementing a series of multifaceted interventions (including suspension of traffic, closures of public venue and home quarantine for all citizen).

Among 473 confirmed COVID-19 cases in Wenzhou, we found that the proportion of asymptomatic patients of COVID-19 was 4.9%, which was higher than those of other researches in the same period [30, 31]. At the same time, 7.6% patients who had no link to Wuhan but contact with individuals from Wuhan without any symptoms at the time of contact. It indicated that the individuals from Wuhan might be the unconfirmed asymptomatic patients with infectious. It is difficult to differentiate and screen patients with atypical symptoms and the rapid human-to-human transmission among close contacts is a critical characteristic for SARS-CoV-2 [7, 32]. Besides, previous studies have reported asymptomatic transmission of SARS-CoV-2 infection through close contacts in both familial and hospital settings [33]. Also, clustered outbreaks caused by asymptomatic individuals were reported [34]. Furthermore, the proportion of patients with undiscovered sources (12.9%) was lower than 25.4% in one of the most recent reports [35], which made it difficult to track close contacts and was not conducive to quickly cutting off the virus transmission route. For transmission involving the patients with unknown sources, there were two likely sources of infection, including indirect transmission and airborne aerosol transmission. The indirect transmission of virus may result from virus contamination of surfaces of objects [36–38]. Aerosol is the small respirable particles that can remain airborne and are capable of short and long‐range transport in several studies [39–43]. For the aerosol and surface stability of virus, SARS-CoV-2 can remain viable and infectious in aerosols for hours and on surfaces up to days, so aerosol and contact transmission of the virus is reasonable [44]. In light of the emergence of unconventional patients, it suggested that cutting off widespread contact transmission and asymptomatic (including unconfirmed asymptomatic patients) transmission should be an important part of COVID-19 epidemic prevention and control (Additional file 3: Fig. S1).

Successful implementation of strict measures requires an unusual and unprecedented speed of decision-making by top leaders, operational thoroughness by public health systems, and cooperation of the whole society in Wenzhou. This study evaluated the epidemic characteristics of Wenzhou after having imported cases from Hubei Province and the outcomes after adopting a series of strict prevention and control measures. In the early stage of the epidemic, COVID-19 cases in Wenzhou was dominated by imported cases, and the number of imported cases peaked on January 21. With the expansion of the epidemic, the number of local cases had increased and became the majority in Wenzhou. However, strict prevention and control measures were not implemented nationwide, nor in Wenzhou on January 21. At this time, Wenzhou had more imported cases, which triggered local transmission in Wenzhou, and was dominated by local cases in the middle and late stages, as indicated in Fig. 2. As the time changed, there was a trend that the epidemic situation shifted from the import stage to the local community spread stage in Wenzhou, and the occurrence of new patients had slowed down, and no case occurred after February 14.

After the outbreak of COVID-19, Wenzhou government launched the first-level response to major public health emergencies on January 23, 2020 [21], and implemented a series of joint prevention and control measures which was focused on preventing importation of cases in order to curb the spread of the disease [18–20]. However, after more local cases occurred, Wenzhou conducted stronger prevention and control measures that were aimed at slowing down the increase in cases and at controlling the import and spread of the epidemic [22, 23], such as closing high-way entrances, limiting the number and frequency of out-of-home activities, regularly releasing updates and information about the epidemic as well as prevention and control measures, strengthening public risk communications and health education, and cancelling mass gathering activities [24–26]. After launching a series of prevention and control measures, the epidemic was effectively controlled. The measures became the normalization mode starting from February 16. The “Wenzhou Health Code” was promoted and applied throughout the city. Villages and communities continued to implement closed-loop management. After the epidemic gradually became under control, the relevant checkpoints were removed and the highway entrances and exits were simultaneously reopened [27–29]. Work and production activities were resumed in Wenzhou. Since January 16, the R_t_ of Wenzhou gradually declined and dropped below 1 on January 30, which then continued to decline, fully proving that the implementation of strict prevention and control measures in Wenzhou had effectively controlled the epidemic spread.

The first outbreak of COVID-19 in Wenzhou was completely kept under control after a series of intensified control interventions. The first confirmed case was reported on January 17, 2020, and a total of 504 confirmed cases were reported as of February 17. A total of 503 cases were discharged and 1 death occurred as of March 16, 2020. Because no new confirmed case occurred in Wenzhou in the 14 days after February 17, the first-level response to emergencies in Wenzhou was adjusted to second-level on March 2. The second-level response to emergencies was adjusted to third-level on March 23 based on the fact that no new local cases occurred in 21 days. During the period after the first COVID-19 epidemic in Wenzhou (February 17 to October 21), a total of 13 imported cases were found in Wenzhou, including 12 cases imported from abroad and 1 from other cities in China. All cases of them were quarantined as they entering Wenzhou and were kept under closed-loop management. No local transmission has occurred up to now, which indicated that the epidemic of COVID-19 in Wenzhou has been under effective control. In the context of the global pandemic of COVID-19 and the possibility of flare-up outbreaks in countries and regions as the northern hemisphere enters the winter season, the mode of COVID-19 control in Wenzhou has a positive reference in the prevention and control of COVID-19.

Our study had some obvious limitations. Firstly, the case information of Wenzhou was extracted from the website of Wenzhou Health Commission, which contained incomplete information (31 cases) in the early stage of the epidemic. Secondly, it is necessary to dynamically observe for a while to determine whether patients were asymptomatic or pre-symptomatic. We did not have dynamic observation therefore we could not distinguish the asymptomatic and pre-symptomatic patients. Lastly, our study was based on case report data, which were more likely to report patients with severe or obvious symptoms, indicating that the proportion of asymptomatic infections was underestimated.

## Conclusions

In conclusion, our study presented that three kinds of unconventional patients in Wenzhou would be an important characteristic of SARS-CoV-2 transmission. The evaluation showed that a series of multifaceted public health control measures had effectively controlled the epidemic of COVID-19. These findings might provide references and experience in the effectiveness of control measures for countries or areas around the world to combat the global pandemic of COVID-19.

## Supplementary Information

**Additional file 1: Appendix S1.** Additional methods.

**Additional file 2: Table S1.** Estimates of the effective reproduction number (R_t_) for laboratory-confirmed COVID-19 cases from January 11 to March 2, 2020.

**Additional file 3: Fig. S1.** The time distribution of cases. The first patient (index patient) with COVID-19 was diagnosed on January 21, 2020 in Wenzhou. Patient X, the earliest date of onset patient, recalled his onset date with symptoms to be January 4, 2020, when he was interviewed by field epidemiologist of WZCDC. The time distribution of all COVID-19 patients was shown by dates of diagnosis in this figure, the majority of the 490 cases (406 cases, 82.9%) occurred during January 27 and February 7, as shown in the diagnosis dates curve. There were four relatively high peaks on January 28, 29, 30, and February 3. The number of cases decreased significantly after that.

## Data Availability

The datasets used and/or analysed during the current study are available from the corresponding author on reasonable request.

## Abbreviations

SARS-CoV-2: Severe acute respiratory syndrome coronavirus 2
COVID-19: Corona virus disease 2019
NCP: Novel coronavirus pneumonia
PHEIC: Public Health Emergency of International Concern
WZCDC: Wenzhou Center for Disease Prevention and Control
R_0_: Basic reproduction number
R_t_: Effective reproduction number
IQR: Interquartile range
