# Supplementary material for: Transmission dynamics of SARS-CoV-2 in a mid-size city of China
Source: BMC Infect Dis. 2021 Aug 10;21:793. doi: 10.1186/s12879-021-06522-9 (PMC8353423; doi:10.1186/s12879-021-06522-9)
Supplement: Supplementary file 1 — Additional file 1: Appendix S1. Additional methods. [file 12879_2021_6522_MOESM1_ESM.docx]

**Additional methods**

**The exposure** history was defined as an individual had the following situations within 14 days before the symptoms onset (1) traveled to or lived in Wuhan or other regions with severe epidemics abroad; (2) had contact with SARS-CoV-2 infected individuals whose nucleic acid test was positive or contact with patients who had a fever or respiratory symptoms coming from Wuhan or other regions abroad that have been severely affected by the epidemic; (3) had been to areas where 2 or more cases developed fever or respiratory symptoms in a small confined area (such as home, office, school class, etc.) [[13](#_ENREF_15),37].

**Cluster outbreak** was defined as the situation that 2 or more cases developed fever or respiratory symptoms in a small area (such as home, office, school class, workshop, construction site, etc.) within 14 days, which considering possible interpersonal transmission and being infectious due to co-exposure [[13](#_ENREF_15),37].

**Close contacts** were defined as people who had close contact (within 1m) with suspected or confirmed cases without effective protection (i.e. wear face mask) 2 days before the onset of symptoms or 2 days before the collection of asymptomatic specimens [[13](#_ENREF_15),37].

**The suspected** case was defined by both epidemiological history and clinical characteristics. An individual was defined as a suspected case with any one type of epidemiological history meets any two of the clinical characteristics; those without a clear epidemiological history meet three of the clinical characteristics. Epidemiological history refers to: (1) History of traveling to or living in Wuhan and surrounding areas, or other communities in China with case reports, or countries and regions with severe epidemics abroad within 14 days before the onset of illness; (2) History of contact with SARS-CoV-2 infected patients (individuals whose nucleic acid test was positive) within 14 days before the onset of illness; (3) History of contact with patients who had a fever or respiratory symptoms, coming from Wuhan and surrounding areas, or other communities with case reports in China, or countries and regions with severe epidemics abroad within 14 days before the onset of illness; (4) Cluster outbreak. Clinical characteristics refer to: (1) Fever and / or respiratory symptoms; (2) Radiologic imaging features of SARS-CoV-2 pneumonia; (3) Total count of white cells was normal or decreased, or the lymphocyte count was normal or decreased in the early stage of onset [[13](#_ENREF_15)].

**Co-exposed person** was defined as an individual who shared the same risks of exposure as a suspected or confirmed case of COVID-19 [[13](#_ENREF_15)].

13. National Health Commission of the People’s Republic of China. New coronavirus pneumonia case surveillance program. 5th ed; 2020. <http://www.nhc.gov.cn>. Assessed 29th February, 2020.

37. Xie C, Zhao H, Li K, [Zhang](javascript:;) Z , Lu X, [Peng](javascript:;) H, et al. The evidence of indirect transmission of SARS-CoV-2 reported in Guangzhou, China. BMC Public Health, 2020;20:1202.

**Intervention and surveillance measures**

**Close-loop management**

Close-loop management, a measure of normalization mode for COVID-19 controlling, regulated that people who were from epidemic-risk areas before 14 days or occurred symptom and their close contacts were required home-quarantine or medical observation after they entering Wenzhou.

**Close-off management**

Close-off management was a stricter measure of COVID-19. When a cluster of cases break out, the community must immediately implement full or partial closed-off management according to the risk level. When one confirmed case occurred, the whole building must implement closed management. During the closed-off management period, cadres of the towns (streets) and villages (communities, residential) were on rotational duty 24 hours a day and were obliged to provide psychological counseling services. Village (resident) residents were in principle not allowed to enter and exit except for special circumstances.

**Application of “Wenzhou Health Code”**

Personnel and three kinds of people who were ready to return to work, return to school or return to Wenzhou could apply online for "Wenzhou Health Code" by themselves. They would open Alipay and search for "Wenzhou Health Code", fill in personal health information, and submit the application after confirmation (after successful application). After application, the "health code" is available in the Alipay "card package" certificate column. Applicants must fill in the information truthfully, and those who fail to fill in factfully could potentially face legal consequences that would be shown in the personal integrity record. Those who caused serious medical consequences would be held accountable in accordance with the law. After the application was reviewed, a three-color "health code" would be generated, including green, yellow and red code. Those who displayed a "green code" and whose temperature measurement was normal were allowed to pass while those who displayed a "yellow code" would be subject to 14-day and 7-day home quarantine observation according to the epidemic-risk areas and low-risk areas respectively. Those that displayed the "red code" were send to designated hospital for treatment or medical isolation and observation for 14 days. Villages (residential areas) and communities were continually implemented closed-loop management in Wenzhou. When entering the implementation of one-way temperature measurement, the village and community personnel shall rely on the “health green code” passes, and those who had not applied for the health code could enter with valid certificates. Individual with a “yellow health code” could enter for the first time, and strictly followed the regulations to implement home isolation observation after entering. The closed-loop measures were implemented by the local township (street) government for open communities without management and security. In key areas for prevention and control, the relevant counties (cities, districts) could appropriately extend the village (resident) travel control measures as deemed appropriate.
