# Supplementary material for: Transmission dynamics of SARS-CoV-2 in a mid-size city of China
Source: BMC Infect Dis. 2021 Aug 10;21:793. doi: 10.1186/s12879-021-06522-9 (PMC8353423; doi:10.1186/s12879-021-06522-9)
Supplement: Supplementary file 2 — Additional file 2: Table S1. Estimates of the effective reproduction number (Rt) for laboratory-confirmed COVID-19 cases from January 11 to March 2, 2020. [file 12879_2021_6522_MOESM2_ESM.docx]

**Additional file 2: Table S1** Estimates of the effective reproduction number (R_t_) for laboratory-confirmed COVID-19 cases from January 11 to March 2, 2020

| Date | Mean | Std | Median | 95% CI |
| --- | --- | --- | --- | --- |
| 2020/1/11 | 3.69 | 1.84 | 3.38 | 1.00~8.08 |
| 2020/1/12 | 2.98 | 1.49 | 2.73 | 0.81~6.53 |
| 2020/1/13 | 3.06 | 1.37 | 2.86 | 0.99~6.26 |
| 2020/1/14 | 2.50 | 1.12 | 2.34 | 0.81~5.13 |
| 2020/1/15 | 3.21 | 1.14 | 3.08 | 1.39~5.79 |
| 2020/1/16 | 3.74 | 1.08 | 3.63 | 1.93~6.13 |
| 2020/1/17 | 3.29 | 0.88 | 3.21 | 1.80~5.22 |
| 2020/1/18 | 2.07 | 0.60 | 2.02 | 1.07~3.40 |
| 2020/1/19 | 1.75 | 0.47 | 1.71 | 0.96~2.78 |
| 2020/1/20 | 1.74 | 0.40 | 1.71 | 1.05~2.60 |
| 2020/1/21 | 1.84 | 0.35 | 1.82 | 1.21~2.59 |
| 2020/1/22 | 2.10 | 0.33 | 2.08 | 1.51~2.79 |
| 2020/1/23 | 1.85 | 0.27 | 1.84 | 1.36~2.41 |
| 2020/1/24 | 1.83 | 0.23 | 1.82 | 1.40~2.31 |
| 2020/1/25 | 1.72 | 0.19 | 1.71 | 1.36~2.12 |
| 2020/1/26 | 1.76 | 0.17 | 1.76 | 1.44~2.12 |
| 2020/1/27 | 1.44 | 0.14 | 1.44 | 1.18~1.73 |
| 2020/1/28 | 1.30 | 0.12 | 1.30 | 1.07~1.54 |
| 2020/1/29 | 1.10 | 0.10 | 1.09 | 0.91~1.30 |
| 2020/1/30 | 0.91 | 0.09 | 0.91 | 0.75~1.09 |
| 2020/1/31 | 0.82 | 0.08 | 0.82 | 0.68~0.98 |
| 2020/2/1 | 0.76 | 0.07 | 0.75 | 0.62~0.90 |
| 2020/2/2 | 0.63 | 0.06 | 0.63 | 0.52~0.76 |
| 2020/2/3 | 0.60 | 0.06 | 0.60 | 0.49~0.72 |
| 2020/2/4 | 0.54 | 0.06 | 0.54 | 0.44~0.65 |
| 2020/2/5 | 0.48 | 0.05 | 0.47 | 0.38~0.59 |
| 2020/2/6 | 0.49 | 0.05 | 0.49 | 0.39~0.60 |
| 2020/2/7 | 0.46 | 0.05 | 0.46 | 0.36~0.57 |
| 2020/2/8 | 0.46 | 0.05 | 0.46 | 0.36~0.58 |
| 2020/2/9 | 0.43 | 0.05 | 0.43 | 0.33~0.54 |
| 2020/2/10 | 0.43 | 0.06 | 0.42 | 0.32~0.54 |
| 2020/2/11 | 0.38 | 0.06 | 0.38 | 0.28~0.50 |
| 2020/2/12 | 0.40 | 0.06 | 0.40 | 0.29~0.53 |
| 2020/2/13 | 0.36 | 0.06 | 0.36 | 0.26~0.49 |
| 2020/2/14 | 0.30 | 0.06 | 0.30 | 0.20~0.43 |
| 2020/2/15 | 0.19 | 0.05 | 0.19 | 0.11~0.30 |
| 2020/2/16 | 0.15 | 0.04 | 0.14 | 0.07~0.25 |
| 2020/2/17 | 0.11 | 0.04 | 0.10 | 0.04~0.20 |
| 2020/2/18 | 0.12 | 0.05 | 0.12 | 0.05~0.23 |
| 2020/2/19 | 0.04 | 0.03 | 0.03 | 0.00~0.11 |
| 2020/2/20 | 0.05 | 0.03 | 0.04 | 0.01~0.13 |
| 2020/2/21 | 0.03 | 0.03 | 0.02 | 0.00~0.11 |
| 2020/2/22 | 0.04 | 0.04 | 0.02 | 0.00~0.13 |
| 2020/2/23 | 0.04 | 0.04 | 0.03 | 0.00~0.17 |
| 2020/2/24 | 0.06 | 0.06 | 0.04 | 0.00~0.21 |
| 2020/2/25 | 0.08 | 0.08 | 0.05 | 0.00~0.28 |
| 2020/2/26 | 0.10 | 0.10 | 0.07 | 0.00~0.38 |
| 2020/2/27 | 0.14 | 0.14 | 0.10 | 0.00~0.52 |
| 2020/2/28 | 0.20 | 0.20 | 0.14 | 0.00~0.73 |
| 2020/2/29 | 0.28 | 0.28 | 0.19 | 0.01~1.02 |
| 2020/3/1 | 0.39 | 0.39 | 0.27 | 0.01~1.44 |
| 2020/3/2 | 0.55 | 0.55 | 0.38 | 0.01~2.03 |

Results were shown since January 11, calculated for the whole period

(from January 11 to March 2) over 7-day moving average.
