# Supplementary figures and images for: Transmission dynamics of SARS-CoV-2 in a mid-size city of China
Source: BMC Infect Dis. 2021 Aug 10;21:793. doi: 10.1186/s12879-021-06522-9 (PMC8353423; doi:10.1186/s12879-021-06522-9)

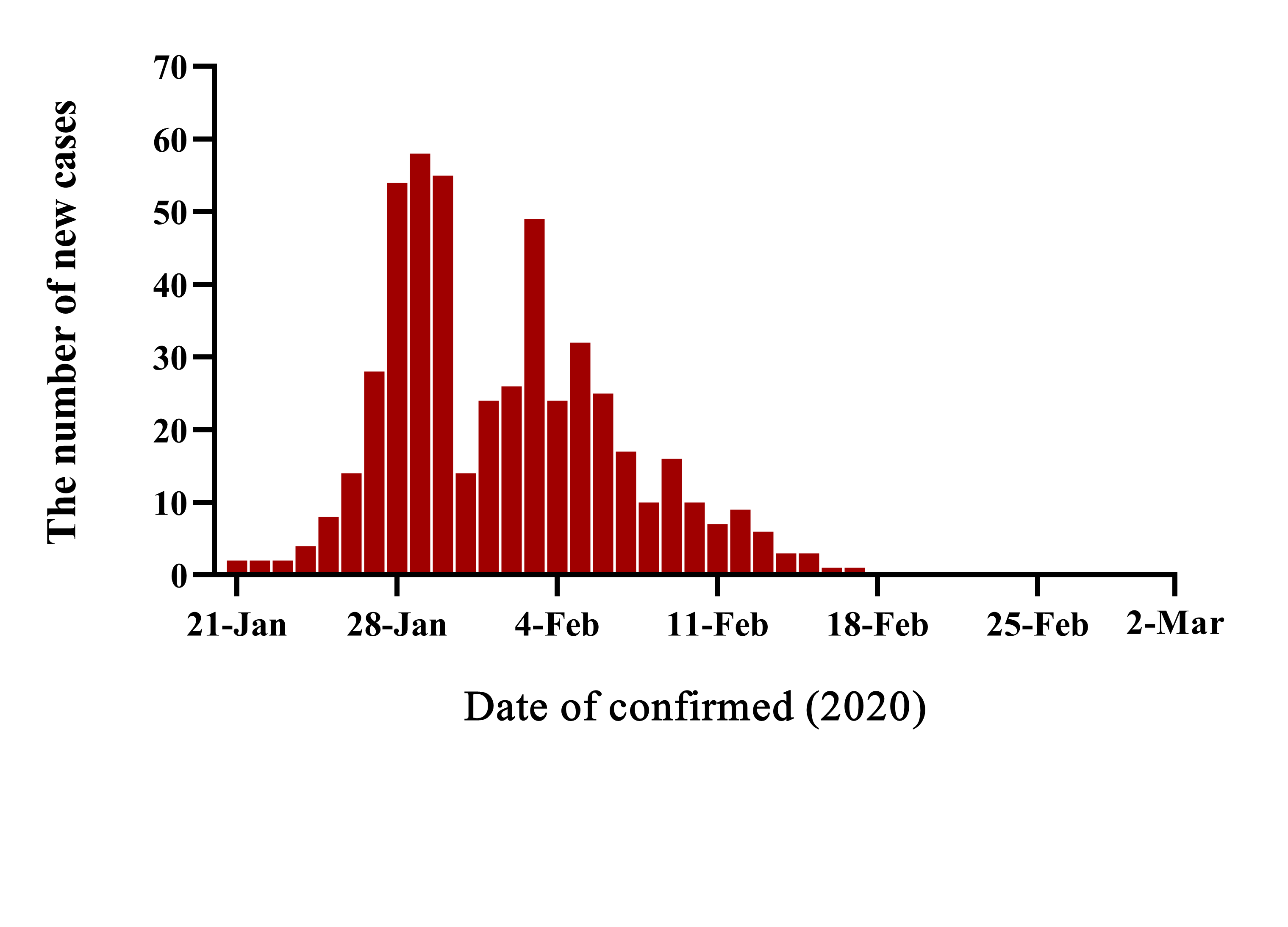

Supplement: Supplementary file 3 — Additional file 3: Fig. S1. The time distribution of cases. The first patient (index patient) with COVID-19 was diagnosed on January 21, 2020 in Wenzhou. Patient X, the earliest date of onset patient, recalled his onset date with symptoms to be January 4, 2020, when he was interviewed by field epidemiologist of WZCDC. The time distribution of all COVID-19 patients was shown by dates of diagnosis in this figure, the majority of the 490 cases (406 cases, 82.9%) occurred during January 27 and February 7, as shown in the diagnosis dates curve. There were four relatively high peaks on January 28, 29, 30, and February 3. The number of cases decreased significantly after that. [file 12879_2021_6522_MOESM3_ESM.png]
